# Gradient-Free Isolation of Murine Pancreatic Islets for Single-Cell RNA Sequencing

**DOI:** 10.3390/mps9050136

**Published:** 2026-09-19

**Authors:** Evgeny S. Ruchko, Zakhar R. Starinnov, Marat S. Sabirov, Maria B. Chernysheva, Arthur A. Lee, Vagif Ali oglu Gasanov, Andrey V. Vasiliev

**Affiliations:** 1 Koltzov Institute of Developmental Biology of the Russian Academy of Sciences, 119334 Moscow, Russia; 2 Department of Embryology, Faculty of Biology, Lomonosov Moscow State University, 119234 Moscow, Russia

**Keywords:** pancreatic islet isolation, mouse model, type 2 diabetes, scRNA-seq

## Abstract

Isolation of pancreatic islets for single-cell RNA sequencing (scRNA-seq) remains technically challenging because enzymatic digestion, mechanical dissociation, and subsequent purification can cause cell loss, aggregation, and processing-induced transcriptional changes. This study aimed to adapt a practical gradient-free alternative for preparing murine pancreatic islets for downstream single-cell transcriptomic analysis while reducing the number of sample-processing steps. The workflow combined intraductal perfusion of the pancreas with 3 mL of a collagenase solution through the common bile duct, controlled enzymatic digestion at 37 °C, performed manual islet selection without density-gradient purification, and achieved gentle dissociation with Accutase and EDTA. DNase I was included in all post-digestion steps to limit cell aggregation. The protocol was evaluated using pancreatic islets from BKS.Cg-Dock7^m^ +/+ Lepr^db^/J mice, a model of type 2 diabetes. Compared with Ficoll-based purification, the gradient-free protocol provided viable cell yields within a similar range while eliminating density-gradient preparation, an 18 min gradient centrifugation step, two subsequent wash centrifugations, and the associated transfer and dilution steps. The resulting scRNA-seq library showed satisfactory quality-control (QC) characteristics and retained the major endocrine populations, including β-, α-, δ-, and PP cells. The gradient-free sample also showed a lower overall stress-response score and lower expression of several immediate early genes, although heat shock- and endoplasmic reticulum (ER) stress-associated genes displayed heterogeneous patterns. These differences were considered descriptive because only one pooled scRNA-seq library was generated for each workflow. These findings support gradient-free isolation as a possible alternative to Ficoll-based purification for preparing murine pancreatic islets for scRNA-seq analysis.

## 1. Introduction

Isolation of pancreatic islets for scRNA-seq is inherently stressful, as enzymatic digestion, mechanical disruption and subsequent handling can impair cell viability and alter the transcriptional state of the preparation. These procedures expose islets to inflammatory, hypoxic, osmotic, oxidative, and ER stress; promote the release of extracellular material; and may activate immediate early genes and heat shock-associated programs [1]. Because scRNA-seq captures transient cell states, even short-lived processing-induced responses can introduce technical artifacts and complicate biological interpretation [2].

For rodent islet isolation, the conceptual basis was established by Lacy and Kostianovsky in 1967, who demonstrated that injection of collagenase-containing Hanks’ solution into the pancreatic ductal system, followed by tissue incubation, could disrupt the acinar compartment and release intact islets for collection [3]. Subsequent rodent protocols retained the same general principle but introduced substantial variation in collagenase formulation, enzyme delivery, digestion time, purification, and post-isolation culture. These parameters require individual optimization because both insufficient and excessive digestion can reduce islet recovery and compromise viability and function [4]. In human pancreatic tissue, Ricordi and colleagues subsequently developed an automated approach using a specialized chamber that combines enzymatic and mechanical dissociation with continuous digestion and simultaneous recovery of released islets [5].

The purification step remains a major source of methodological variability. Buemi et al. compared continuous purification with the COBE 2991 processor and discontinuous bottle-based purification and found similar islet yields, 76,292.5 ± 40,550.44 and 79,625 ± 41,484.46 pancreatic islets, respectively (*p* = 0.89), as well as similar stimulation indices, 3.31 ± 0.83 and 5.58 ± 3.38 (*p* = 0.22). However, the bottle method yielded fewer islets smaller than 100 µm and a greater proportion of islets in the 200–250 µm range, which the authors attributed to reduced shear stress during processing [6]. These findings indicate that purification may influence islet morphology even when overall yield and secretory function remain similar. Density-gradient purification also does not necessarily produce a completely endocrine preparation. Mita et al. reported ductal-cell fractions of 18.5 ± 12.7% and 11.5 ± 6.1% after OptiPrep- and Ficoll-based purification, respectively [7]. Thus, residual non-endocrine cells may remain even after gradient separation. Mechanical mincing followed by enzymatic digestion in suspension represents an alternative to intraductal perfusion. Alternative rodent protocols based on direct pancreatic digestion and Percoll purification have also been reported to provide high islet yields while preserving viability and functional responses similar to those obtained after common bile duct perfusion. However, such methods may require longer digestion periods and may initially contain a larger exocrine fraction [8]. For this reason, most protocols include density-gradient purification using Ficoll, Histopaque, Percoll, dextrans, iodixanol, or combinations of these media. Comparative studies suggest that Ficoll- and Histopaque-based gradients provide a favorable balance of purity, viability, and functional preservation relative to several alternative solutions [9].

Despite these advantages, density-gradient purification adds centrifugation, transfer, and washing steps that prolong sample handling and may expose islets to additional mechanical and osmotic stress. Differences between purification media have also been associated with changes in cytokine and chemokine production and in β-cell survival during subsequent culture, indicating that purification may influence the biological state of the preparation even when conventional measures of yield and viability remain similar [7]. Processing time represents an additional source of variability. In a 24 h scRNA-seq time-course study of human islets, Grenko et al. identified 1311 genes associated with time in culture and 345 genes associated with glucose exposure, demonstrating that transcriptional changes accumulate even during relatively short ex vivo incubation [10]. Moreover, direct comparison of scRNA-seq and single-nucleus RNA sequencing data from the same donors confirmed that single-cell dissociation can introduce stress-associated transcriptional artifacts and alter the recovery of individual cell populations [11].

Following pancreatic digestion, islets can be enriched by density-gradient purification, stereomicroscope-guided manual selection, or combinations of these approaches, each requiring different degrees of centrifugation, washing, transfer, and handling before dissociation [4]. These pre-analytical differences are particularly relevant for molecular studies because the cellular transcriptome can be affected by tissue dissociation and ex vivo processing. More broadly, molecular and epigenetic alterations are increasingly being investigated as clinically relevant biomarkers in pancreatic disease, particularly pancreatic cancer. Aberrant DNA methylation, histone modifications, and dysregulated non-coding RNAs have been associated with diagnosis, prognosis, and therapeutic responses, highlighting the importance of standardized sample procurement and processing for reproducible molecular measurements [12].

Together, these findings support minimizing the interval between pancreas collection, islet purification, dissociation, and library preparation. A dedicated mouse islet protocol described by Lee and Engin for scRNA-seq combines common bile duct perfusion, Histopaque purification, manual selection, overnight recovery, and Accutase dissociation, consistently producing single-cell suspensions with approximately 90% viability [13]. Although effective, this workflow involves multiple centrifugation, washing, purification, and culture steps before library preparation. Methanol fixation has emerged as a practical approach for preserving dissociated cells before scRNA-seq library preparation, thereby enabling flexible sample handling while maintaining RNA integrity [14].

**Figure 1:**
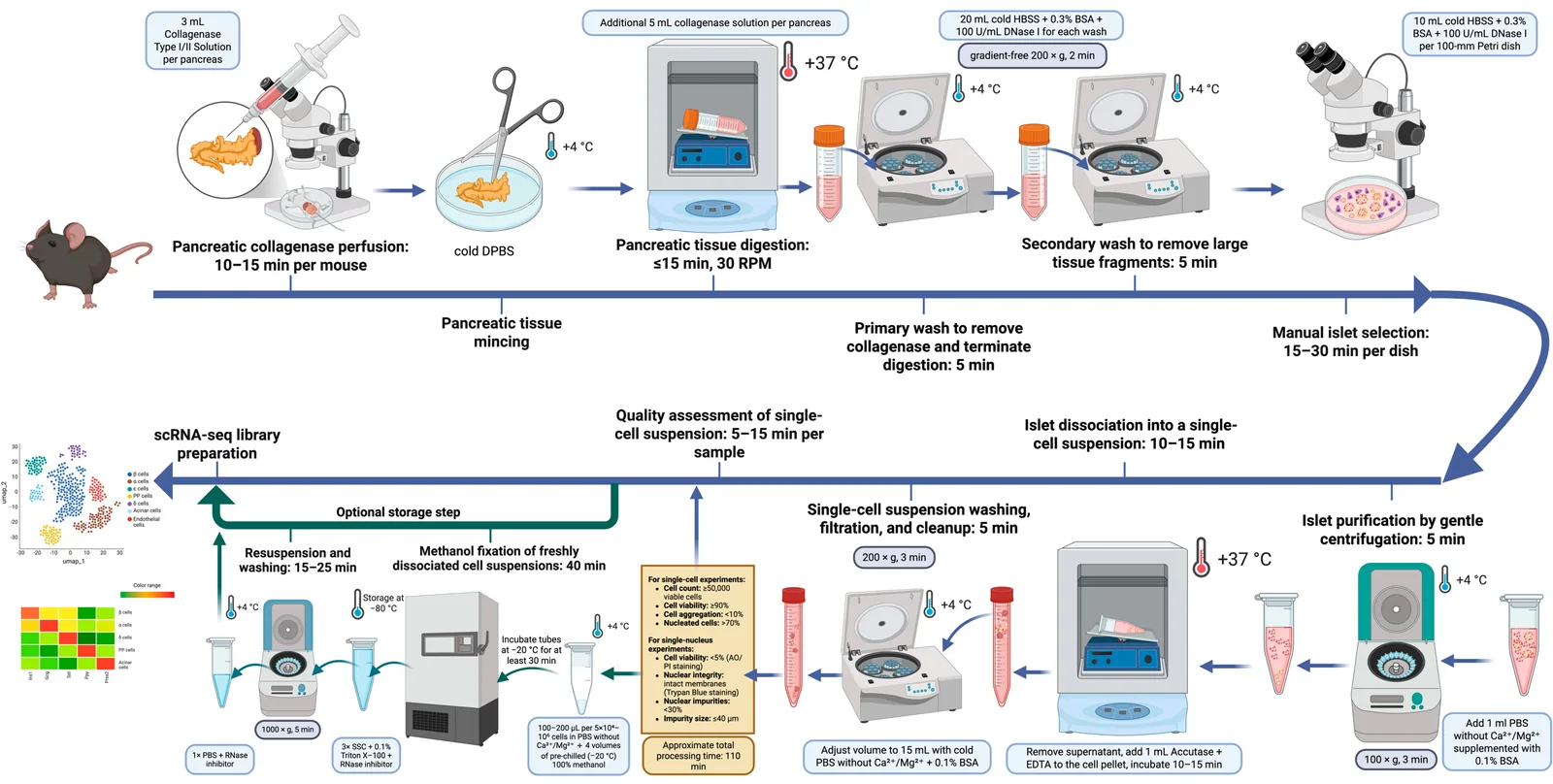
Workflow for gradient-free pancreatic islet isolation and scRNA-seq preparation. The protocol begins with intraductal collagenase perfusion of the pancreas through the common bile duct, followed by pancreatic excision, tissue mincing, controlled collagenase digestion, and termination of enzymatic activity by cold washing. After removal of large tissue fragments, pancreatic islets are manually selected under a stereomicroscope and gently pelleted before dissociation into single cells using Accutase/EDTA. The resulting suspension is washed, filtered, and subjected to QC prior to library preparation. An optional methanol fixation step enables storage of dissociated cells before scRNA-seq. Approximate processing times, temperatures, centrifugation conditions, and reagent volumes are indicated for each step. Created in BioRender. Ruchko, E. (2026) https://BioRender.com/qmheuti.

Accordingly, the present study aimed to evaluate a gradient-free pancreatic islet isolation workflow for BKS.Cg-Dock7^m^ +/+ Lepr^db^/J mice for subsequent scRNA-seq analysis (Figure 1). The protocol was designed to omit Ficoll-based density-gradient purification and reduce the number of Ficoll-specific centrifugation, washing, and transfer steps before single-cell preparation. Its feasibility for scRNA-seq was assessed based on cell recovery and viability, library QC characteristics, cell-type representation, and descriptive analysis of stress-associated transcriptional features. Manual islet selection, controlled enzymatic digestion, and gentle dissociation were used to obtain a sufficient number of viable cells while retaining the major endocrine populations. Relative to Ficoll-based purification, the gradient-free approach eliminated density-gradient preparation, interphase recovery, and two subsequent washing steps. This removed three centrifugation steps and shortened the estimated overall processing time by approximately 35 min, including 21.5 min of centrifugation, while providing sufficient viable cells for scRNA-seq library preparation. The aim was therefore to evaluate the gradient-free workflow as a practical alternative to Ficoll-based purification rather than to establish its superiority.

## 2. Materials and Methods

### 2.1. Animals, Experimental Design and Reagent Preparation

Pancreatic islets were isolated from male BKS.Cg-Dock7^m^ +/+ Lepr^db^/J mice aged 4.5 months. The mean body weight of the animals was 40 ± 3 g and consistently elevated blood glucose concentrations ranging from 15 to 20 mmol/L were observed. For each isolation protocol, pancreatic islets were obtained from three mice in three independent isolation procedures, with one mouse processed per isolation. Cell yield and viability were recorded separately for each animal. Following these measurements, material from the three independent isolations was pooled to generate one scRNA-seq library for each protocol. All animal procedures were approved by the Institutional Ethics Committee of the Koltzov Institute of Developmental Biology of the Russian Academy of Sciences (Protocol No. 94, dated 5 June 2025).

Before pancreatic islet isolation, all reagents and consumables were prepared in advance. A collagenase solution containing 3 mg/mL collagenase type I (act: 270 U/mg, Lot. 44N24865, Worthington Biochemical, Lakewood, NJ, USA) and 3 mg/mL collagenase type II (act: 340 U/mg, Lot. 41B20909, Worthington Biochemical, USA) was prepared in Dulbecco’s phosphate-buffered saline (PanEco, Moscow, Russia) at a volume of 8 mL per animal. DNase I (Thermo Fisher Scientific, Waltham, MA, USA) was added to a final concentration of 100 U/mL. The solution was drawn into syringes and kept on ice until use. Hanks’ balanced salt solution (PanEco, Russia) supplemented with 0.3% bovine serum albumin (BSA) (Sigma-Aldrich, St. Louis, MO, USA) and DNase I at 100 U/mL, as well as Roswell Park Memorial Institute 1640 (RPMI 1640) medium (PanEco, Russia) containing 0.3% BSA, was also pre-cooled on ice. The centrifuge was cooled to 4 °C, and sterile 50 mL conical tubes and 100 mm Petri dishes were prepared in advance.

All pancreatic islet isolations and stereomicroscope-guided manual selection procedures were performed by the same trained operator who had experience with cell isolation procedures involving different cell types. A complete list of reagents and materials used for pancreatic islet isolation, single-cell preparation, and scRNA-seq library construction is provided in Appendix A (Table A1).

### 2.2. Pancreatic Perfusion, 10–15 min per Mouse

Before pancreatic perfusion, mice were anesthetized with tribromoethanol (Avertin, Labtech Ltd., Moscow, Russia) administered intraperitoneally at a dose of 250 mg/kg. A 2.5% working solution was used, corresponding to an injection volume of 10 µL/g body weight. After confirmation of deep anesthesia, the subsequent procedure was performed in accordance with the approved institutional animal protocol. Pancreatic perfusion and tissue collection were performed as a terminal procedure, and animals were euthanized by cervical dislocation while under deep anesthesia.

**Figure 2:**
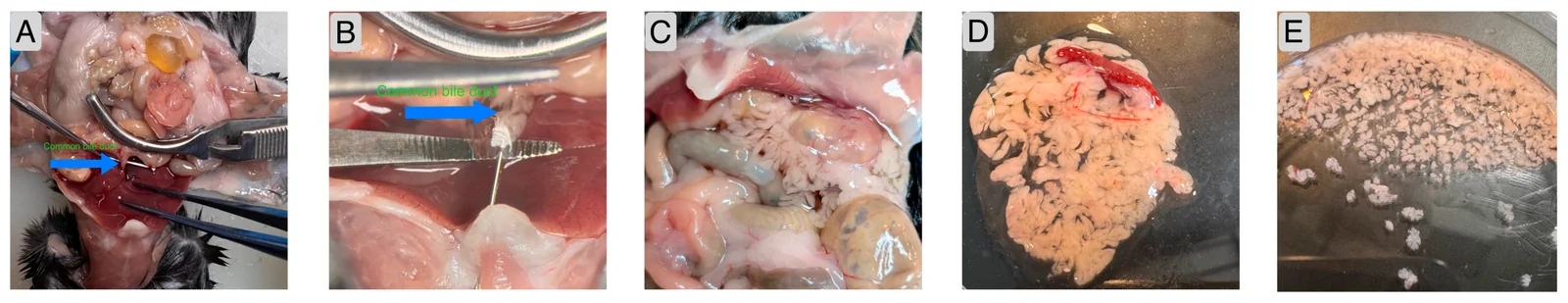
Pancreatic perfusion and assessment of collagenase delivery. (**A**) Cannulation of the common bile duct and intraductal perfusion of the mouse pancreas with the collagenase type I/II solution. (**B**) Insertion of a 30G needle into the common bile duct for collagenase injection. (**C**) Example of successful pancreatic perfusion. (**D**) Gross appearance of a successfully perfused pancreas after excision. Uniform lobular expansion confirms adequate distribution of collagenase throughout the tissue before enzymatic digestion. (**E**) Excised collagenase-perfused pancreas before tissue digestion. Uniform separation of the pancreatic lobules confirms successful intraductal perfusion and adequate enzyme penetration.

After opening the abdominal cavity, the common bile duct was identified, and a Bulldog vascular clamp was placed to occlude the ampulla of Vater (Figure 2A). A 30G needle connected to a syringe containing a cold collagenase solution was then inserted into the duct from the proximal branching region (Figure 2B). The pancreas was perfused with 3 mL of the collagenase solution until uniform tissue inflation was achieved (Figure 2C). The pancreas was carefully excised and immediately transferred to a dish or tube containing cold Dulbecco’s phosphate-buffered saline on ice (Figure 2D).

### 2.3. Primary Pancreatic Digestion, up to 15 min

**Figure 3:**
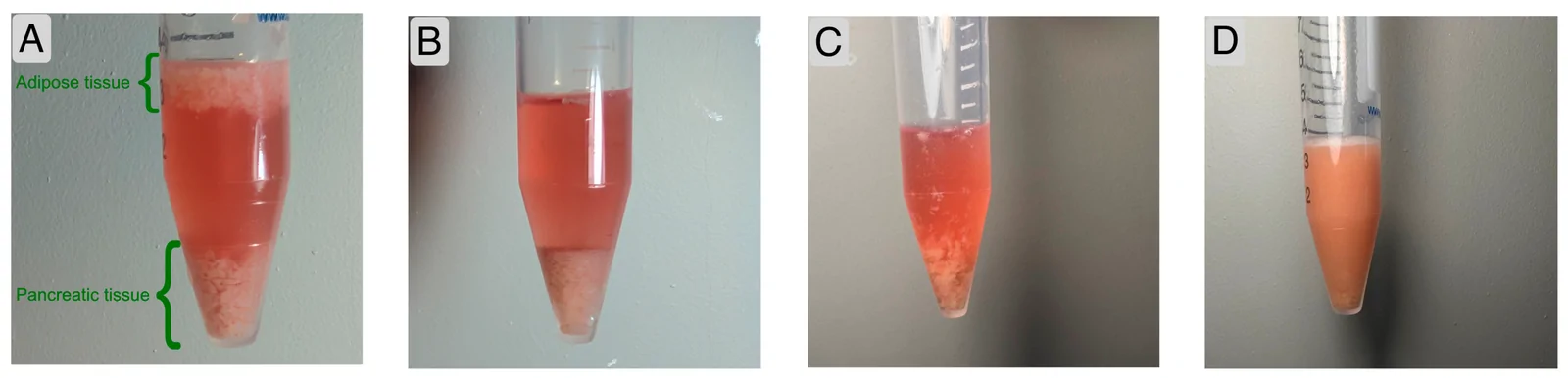
Visual assessment of pancreatic tissue digestion with collagenase. (**A**) Pancreatic tissue suspension after excision, showing floating adipose tissue in the upper layer and pancreatic tissue at the bottom of the tube. (**B**) Pancreatic tissue suspension before incubation with collagenase. (**C**) Example of insufficient collagenase digestion. Large, incompletely dissociated tissue fragments remain visible at the bottom of the tube. (**D**) Example of adequate collagenase digestion. The pancreatic tissue is uniformly fragmented, forming a fine suspension suitable for subsequent washing and manual islet selection.

The pancreas was minced with scissors and transferred to a 50 mL tube containing 5 mL of the collagenase solution (Figure 2E). The tissue was incubated at 37 °C for no longer than 15 min on an MR-1 Mini-Rocker Shaker (BioSan, Riga, Latvia) at 30 rpm using heat-insulating beads, placed inside an MCO-170AC-PE CO_2_ Incubator, and maintained at 37 °C (PHCbi (PHC Corporation), Tokyo, Japan) (Figure 3). Tissue softening and fragmentation were assessed visually every 5 min. Digestion was stopped once the pancreas became fragmented and readily dissociated during gentle mixing.

### 2.4. Termination of Digestion and Primary Washing, 5 min

The tube was removed from the incubator and transferred to a cell-culture laminar-flow cabinet. Twenty milliliters of cold Hanks’ balanced salt solution (HBSS) (PanEco, Russia) containing 0.3% BSA and 100 U/mL DNase I was added, and the suspension was gently mixed by inversion. DNase I was used at all stages following enzymatic tissue disruption. The suspension was centrifuged at 200× *g* for 2 min at 4 °C using an Eppendorf Centrifuge 5810 R (Eppendorf, Hamburg, Germany) equipped with an A-4-81 swing-bucket rotor. The supernatant was carefully removed, leaving approximately 1 mL above the pellet.

### 2.5. Removal of Large Tissue Fragments and Additional Washing, 5 min

The pellet was resuspended in 10 mL of cold HBSS containing 0.3% BSA and 100 U/mL DNase I using 1000 µL wide-bore pipette tips (GenFollower Biotech Co., Ltd., Shaoxing, China, distal opening diameter, 2.05 mm). The suspension was transferred to a new 50 mL tube while avoiding the transfer of large tissue fragments and floating adipose tissue. The original tube was rinsed with an additional 10 mL of HBSS containing 0.3% BSA and 100 U/mL DNase I. The wash was combined with the main suspension, which was then gently pipetted 5–7 times. The suspension was centrifuged at 200× *g* for 2 min at 4 °C using an Eppendorf Centrifuge 5810 R equipped with an A-4-81 swing-bucket rotor (Eppendorf SE, Hamburg, Germany). The supernatant was removed, and the pellet was carefully resuspended in 10 mL of HBSS containing 0.3% BSA and 100 U/mL DNase I.

### 2.6. Ficoll-Based Islet Purification, 35 min

Commercially prepared Ficoll solutions (PanEco, Russia) with densities of 1.100 and 1.077 g/mL were used as supplied by the manufacturer, without further dilution or density adjustment, and were pre-cooled to 4 °C before use.

For the Ficoll-based purification protocol, approximately 100 µL of supernatant was left above the pellet after the initial wash. The pellet was gently resuspended in 5 mL of cold Ficoll with a density of 1.100 g/mL, followed by an additional 5 mL being added along the tube wall to recover residual islets. Ficoll with a density of 1.077 g/mL and then cold RPMI 1640 were carefully layered on top without mixing. The gradient was centrifuged at 900× *g* for 18 min at 4 °C with the brake disabled using an Eppendorf Centrifuge 5810 R equipped with an A-4-81 swing-bucket rotor. The islet-enriched interphase was collected into a new 50 mL tube, diluted to 50 mL with cold RPMI 1640, and washed twice at 200× *g* and 4 °C for 2 min and 90 s, respectively, using an Eppendorf Centrifuge 5810 R equipped with an A-4-81 swing-bucket rotor. The final pellet was resuspended in RPMI 1640 for further processing. This purification step was omitted in the gradient-free protocol.

### 2.7. Manual Islet Selection, 15–30 min per Dish

**Figure 4:**
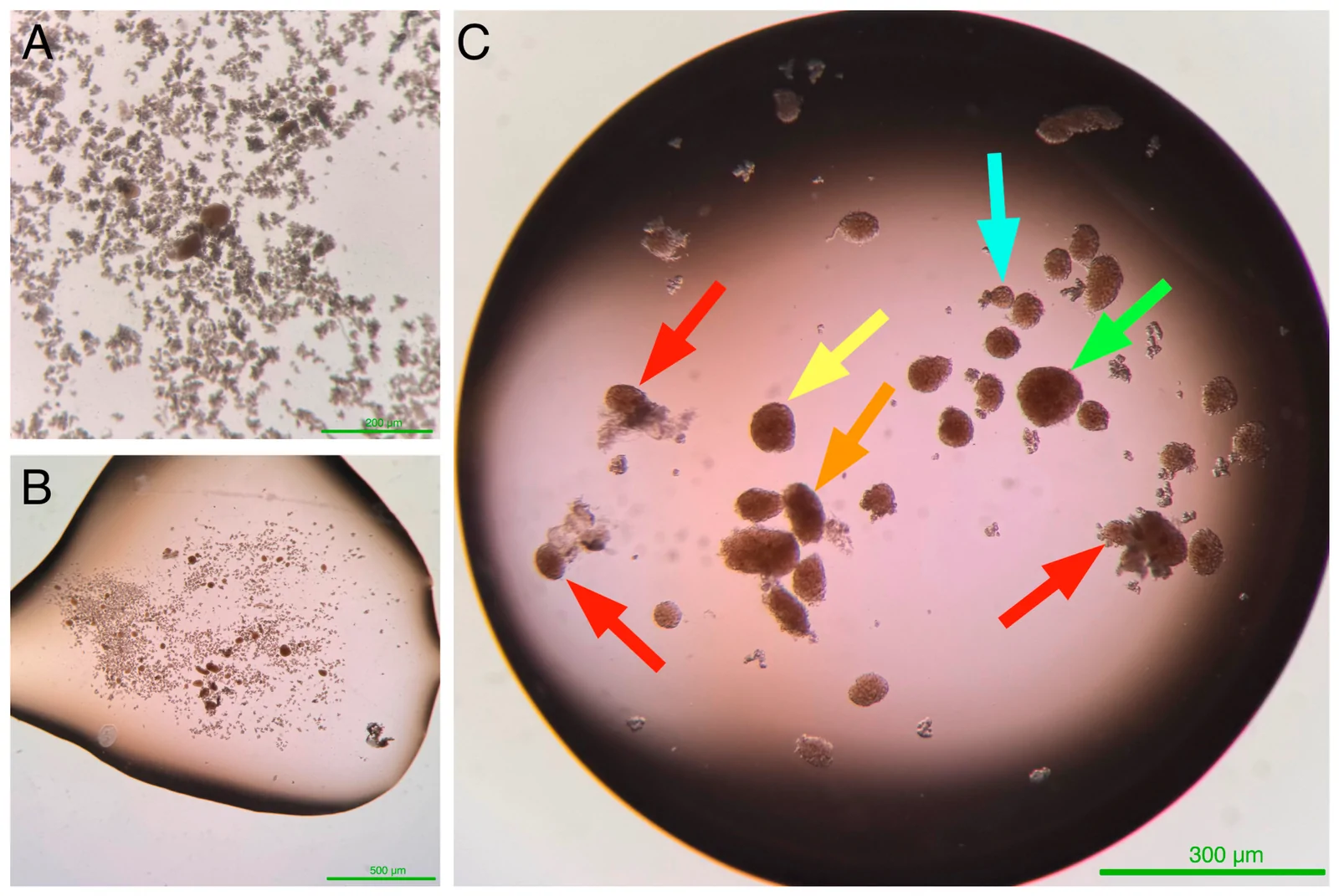
Stereomicroscope-guided manual selection of pancreatic islets. (**A**) Initial pancreatic tissue suspension containing islets among abundant exocrine tissue. (**B**) Intermediate washing step in which manually selected islets are transferred into a clean buffer drop to remove residual tissue fragments. (**C**) Representative final field illustrating the range of islet sizes and morphologies considered during manual selection. The green arrow indicates a large, round islet free of visible acinar tissue; the yellow arrow indicates a medium-sized round islet; the blue arrow indicates a small islet; and the orange arrow indicates a medium-sized elongated islet. Red arrows indicate medium-sized islets associated with residual acinar tissue, which require additional gentle pipetting to facilitate separation before final transfer.

Manual selection was performed under a stereomicroscope placed inside a laminar-flow cabinet after surface disinfection. A 100 mm Petri dish was used, and three 200 µL drops of HBSS containing 0.3% BSA and 100 U/mL DNase I were placed on the inner surface of the lid. Pancreatic islets were collected under the stereomicroscope and transferred into the first drop (Figure 4). After all islets had been selected from the 100 mm dish, they were sequentially transferred from the first drop to the second and then to the third. At each transfer, the islets were gently pipetted two to three times without introducing bubbles to remove residual exocrine tissue. Islets from the third drop were transferred into a 1.5 mL microcentrifuge tube.

No predefined size cutoff was applied during selection. All visually identifiable islets were considered for collection, including small, medium, and large islets, as well as round, elongated, and partially disrupted structures, when present (Figure 4C). Islets associated with adherent acinar tissue were first gently pipetted directly in the Petri dish to remove surrounding exocrine tissue. If the islet could not be sufficiently separated from the surrounding exocrine tissue by gentle pipetting, it was not transferred to the final preparation. During selection, the contents of the dish were gently redistributed every few minutes, after which the entire dish was re-examined under the stereomicroscope to reduce the likelihood of overlooking small or initially obscured islets.

### 2.8. Preparation for Single-Cell Dissociation by Gentle Centrifugation, 5 min

One milliliter of phosphate-buffered saline (PBS) without Ca^2+^ and Mg^2+^ (PanEco, Russia) supplemented with 0.1% BSA was added to the collected islets. The suspension was centrifuged at 100× *g* for 3 min at 4 °C, and the supernatant was carefully removed.

### 2.9. Enzymatic Dissociation of Islets into a Single-Cell Suspension, 10–15 min

For up to 300 islets, 1 mL of Accutase containing EDTA (STEMCELL Technologies, Vancouver, BC, Canada) was used. The islets were washed and centrifuged at 100× *g* for 3 min using an Eppendorf Centrifuge 5415 R equipped with an FA-45-24-11 rotor (Eppendorf SE, Hamburg, Germany), and the supernatant was removed. One milliliter of Accutase with EDTA pre-warmed to 37 °C was then added. The islets were incubated at 37 °C for 10–15 min on an MR-1 Mini-Rocker Shaker at 30 rpm using heat-insulating beads, placed inside an MCO-170AC-PE CO_2_ Incubator, and maintained at 37 °C. Every 3 min, the suspension was gently pipetted using a 200 µL wide-bore tip (GenFollower, China, distal opening diameter, 1.50 mm) to promote gradual dissociation into single cells. The degree of dissociation was monitored microscopically by transferring a 10 µL aliquot to a separate Petri dish. Once a sufficient proportion of single cells had been obtained, the reaction was immediately stopped by adding 500 µL of PBS without Ca^2+^ or Mg^2+^ but supplemented with 0.1% BSA and pre-cooled to 4 °C.

### 2.10. Washing and Concentration of the Single-Cell Suspension, 5 min

The suspension volume was adjusted to 15 mL with cold PBS without Ca^2+^ or Mg^2+^ but supplemented with 0.1% BSA. Cells were centrifuged at 200× *g* for 3 min at 4 °C using an Eppendorf Centrifuge 5810 R equipped with an A-4-81 swing-bucket rotor (Eppendorf SE, Hamburg, Germany). The resulting suspension was passed through a 40 µm cell strainer and washed twice with PBS containing 0.04% BSA without Ca^2+^ or Mg^2+^. The supernatant was carefully removed without disturbing the pellet, and the cells were resuspended in 1 mL of PBS without Ca^2+^ or Mg^2+^.

### 2.11. QC and Preparation for scRNA-seq, 5–15 min per Sample

An aliquot of the cell suspension was used to assess cell concentration and viability by Acridine Orange (Sigma-Aldrich, St. Louis, MO, USA) and Propidium Iodide (Wuhan Servicebio Technology Co., Ltd., Wuhan, China) (AO/PI) fluorescent staining and automated cell counting. Samples with viability of at least 90%, low aggregation, and no fewer than 5 × 10^4^ cells per sample were considered preferable for downstream analysis. The cell concentration was adjusted to the range required for the scRNA-seq platform, typically 700–1200 cells/µL. The suspension was maintained on ice until platform loading, and waiting time and additional handling were minimized. Alternatively, samples were immediately processed using the methanol-fixation protocol for long-term storage at −80 °C.

### 2.12. Methanol Fixation of Dissociated Cells Before scRNA-seq Library Preparation, 40 min

Cells were centrifuged at 200× *g* for 3 min at 4 °C using an Eppendorf Centrifuge 5415 R equipped with an FA-45-24-11 rotor. The supernatant was removed, and the pellet was resuspended in PBS without Ca^2+^ or Mg^2+^ pre-cooled to 4 °C. The volume of the cell suspension before fixation was at least 100 µL. Suspensions containing 5 × 10^3^ to 5 × 10^5^ cells were resuspended in a 100 µL solution, whereas suspensions containing 5 × 10^5^ to 1 × 10^6^ cells were resuspended in a 200 µL solution. The tubes were kept on ice. Four volumes of 100% methanol pre-cooled to −20 °C were added dropwise to the chilled cell suspension with gentle mixing. The samples were mixed carefully by pipetting while avoiding bubble formation and incubated at −20 °C for at least 30 min. Fixed cells were stored at −80 °C for long-term storage of up to 2 months.

### 2.13. Washing of Fixed Cells, 10 min

An appropriate volume, either 500 or 1000 µL, of the fixed-cell suspension was transferred to a new 1.5 mL tube pre-cooled on ice. The amount transferred was calculated to provide 500–12,000 viable cells, as determined before fixation, for preparation of one scRNA-seq library. One milliliter of chilled wash buffer containing 3× saline–sodium citrate buffer (Sigma-Aldrich, USA), 0.1% Triton X-100 (AppliChem GmbH, Darmstadt, Germany), and the RNase inhibitor “RiboCare” (Evrogen, Moscow, Russia) at a final concentration of 1 U/µL was added. The suspension was mixed gently by pipetting and centrifuged at 1000× *g* for 5 min at 4 °C using an Eppendorf Centrifuge 5415 R equipped with an FA-45-24-11 rotor. The supernatant was carefully removed, leaving no more than 5 µL above the pellet.

### 2.14. Resuspension and Preparation for scRNA-seq, 5–15 min

The required volume of the fixed-cell suspension was calculated according to the target cell number for downstream analysis. For preparation of one scRNA-seq library, the final cell concentration was adjusted to 700–1200 cells/µL, with 500–12,000 viable cells, as determined before fixation. The pellet was gently resuspended in 50–300 µL of chilled resuspension buffer consisting of 1× PBS without Ca^2+^ or Mg^2+^ but supplemented with an RNase inhibitor at a final concentration of 1 U/µL. The tube was kept on ice. Cell concentration was reassessed and adjusted when necessary to the range recommended for the scRNA-seq platform.

### 2.15. Cell Concentration and Viability

Cell concentration and viability were determined using a LUNA-FX7 automated fluorescence cell counter (Logos Biosystems, Anyang, Republic of Korea) with AO/PI staining according to the manufacturer’s instructions. Acridine Orange stains nucleated cells, whereas Propidium Iodide selectively labels membrane-compromised (non-viable) cells. Total cell number, viable cell number, cell concentration, and viability were recorded for each preparation before scRNA-seq library construction.

### 2.16. scRNA-seq Library Preparation and Sequencing

All scRNA-seq libraries analyzed in this study were prepared from methanol-fixed and rehydrated single-cell suspensions using the SeekOne Single-Cell 3′ Gene Expression Library Preparation Kit (Beijing SeekGene BioSciences Co., Ltd., Beijing, China) according to the manufacturer’s instructions. Cell suspensions were adjusted to 1200 viable cells/µL based on AO/PI fluorescence counting. Cell viability was 91.2% for the Ficoll-based preparation and 93.4% for the gradient-free preparation. For each sample, 20 µL of the cell suspension, corresponding to approximately 24,000 viable cells, was used for loading onto the SeekOne^®^ DD Chip S3 (Beijing SeekGene BioSciences Co., Ltd., Beijing, China).

The two study libraries were included in a pool of eight indexed libraries and sequenced in a single run on an Illumina NovaSeq 6000 system (Illumina, Inc., San Diego, CA, USA). Sequencing was performed in paired-end mode, generating a 29 bp Read 1 and a 90 bp Read 2. The run generated approximately 3.3–4.1 billion reads in total. Sequencing and mapping quality metrics for both libraries are summarized in Table 1. Although sequencing depth and saturation differed between the libraries, the remaining sequencing and mapping quality metrics were broadly similar.

### 2.17. Bioinformatic Processing and Cell-Type Annotation

Raw sequencing data were processed using SeekSoul v1.2.2. [15] against the GRCm39 reference to generate gene-by-cell count matrices. Downstream analysis was performed in Python using Scanpy v1.12.1 [16]. The gradient-free library yielded 5568 initially identified cells with a mean sequencing depth of 46,913 reads per cell, whereas the Ficoll-based library contained 4358 initially identified cells with 87,251 reads per cell. The median number of detected genes per cell was 1770 and 2532, respectively.

Quality-control filtering was performed separately for each library and followed a defined sequence. Cells with fewer than 200 detected genes, fewer than 500 total transcripts, or mitochondrial transcript content exceeding 10% were excluded from downstream analysis. Putative doublets were identified separately within each library using Scrublet [17] as implemented in Scanpy, with an expected doublet rate of 0.05, a simulated-to-observed doublet ratio of 2.0, 30 principal components, and an automatically determined doublet-score threshold. Ambient RNA correction was performed separately for each library using the remove background module of CellBender [18] with the full noise model, 150 training epochs, a target false-positive rate of 0.01, and a learning rate of 1 × 10^−4^. Following completion of the QC and preprocessing steps, 2659 cells from the gradient-free library and 2715 cells from the Ficoll-based library were retained for downstream analysis. The median number of detected genes per cell was 3480 and 4161, respectively, while the median mitochondrial transcript fraction remained low (1.89% and 1.94%). The distributions of transcript complexity and mitochondrial content broadly overlapped between the two libraries. Predicted doublets were excluded from downstream analysis. The final analyzed dataset contained 2715 cells from the Ficoll-based library and 2659 cells from the gradient-free library (Table 2).

Gene-expression matrices were library-size-normalized and log-transformed. A set of 2000 highly variable genes was retained for downstream dimensionality reduction and clustering using Scanpy. Principal component analysis was performed on the highly variable genes, followed by construction of a nearest-neighbor graph and Uniform Manifold Approximation and Projection (UMAP) [19] for visualization. Cell clusters were identified using the Leiden [20] algorithm (resolution = 1) and annotated based on the expression of established pancreatic endocrine and exocrine marker genes.

Expression patterns of selected marker and stress-response genes were compared descriptively between the Ficoll-based and gradient-free libraries using feature plots, violin plots, and dot plots. No formal differential gene-expression testing was performed between the two libraries. A stress-response score was calculated using the Scanpy sc.tl.score_genes function with the gene set *Fos*, *Fosb*, *Jun*, *Junb*, *Jund*, *Atf3*, *Egr1*, *Dusp1*, *Hspa1a*, *Hspa1b*, *Hsp90aa1*, *Hspb1*, *Ddit3*, *Atf4*, *Xbp1*, *Hmox1*, and *Sod2*. The reference gene set was selected automatically by Scanpy from genes with comparable average expression.

### 2.18. Statistical Analysis

Cell yield and viability were assessed in three independent mouse-derived preparations for each isolation workflow. Each preparation was measured in technical triplicate. Technical replicate measurements were averaged to obtain one value for each mouse, and these per-animal values were treated as independent biological replicates. Data are presented as individual biological replicate values together with the mean ± standard deviation (SD), with n = 3 mice per workflow. Given the small number of biological replicates, comparisons were considered descriptive, and no inferential statistical tests or *p*-values were reported.

For scRNA-seq analysis, material from the three independently processed mice was pooled to generate one library for each isolation workflow. Accordingly, each pooled library represented a single experimental unit, and individual cells were not treated as independent biological replicates. Library QC metrics, relative cell-type composition, gene-expression distributions, and stress-response scores were therefore summarized descriptively. No hypothesis tests, *p*-values, or multiple-comparison corrections were applied to comparisons between the Ficoll-based and gradient-free libraries.

## 3. Results

### 3.1. QC of Single-Cell Suspensions

Omission of Ficoll-based purification removed the preparation and layering of the density gradient, collection of the islet-enriched interphase, dilution to 50 mL, and two subsequent washing steps. The Ficoll-based module required one 18 min gradient centrifugation step and two additional centrifugations of 2 min and 90 s. Together with approximately 15 min of Ficoll-specific handling, this purification module added approximately 35 min to the overall processing time, including 21.5 min of centrifugation.

**Figure 5:**
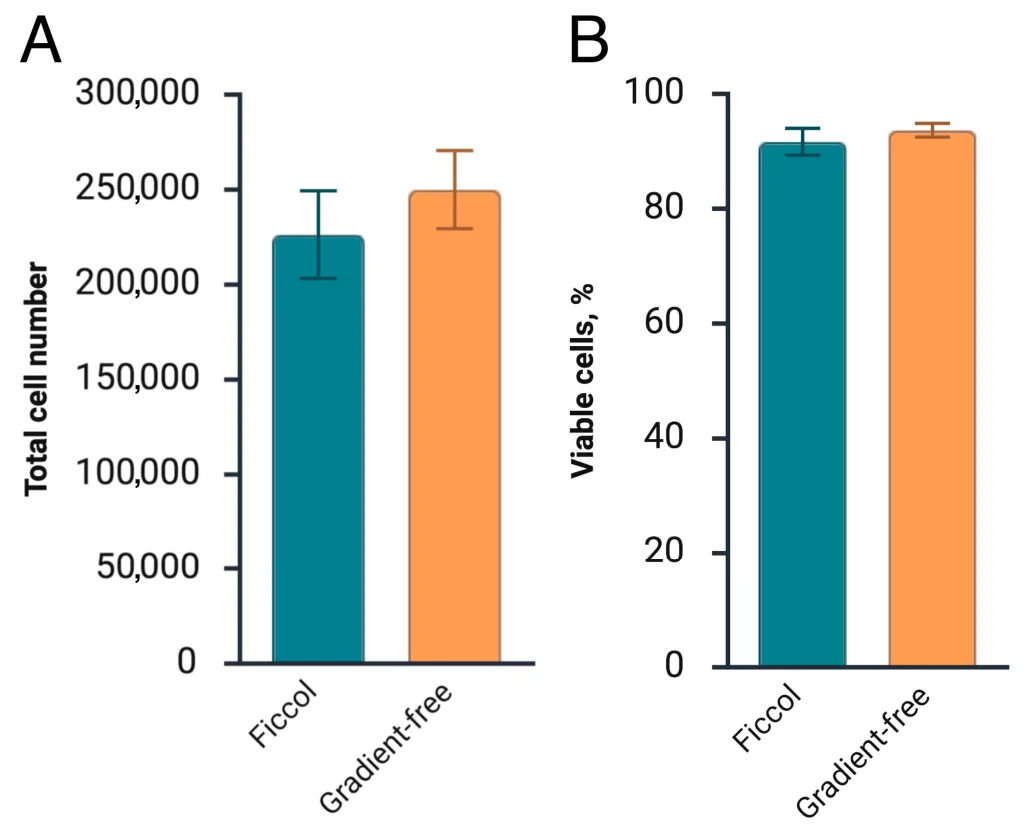
QC of single-cell suspensions before scRNA-seq library preparation. (**A**) Total number of cells obtained after dissociation using the Ficoll-based and gradient-free workflows. (**B**) Cell viability (%) after dissociation. Bars represent the mean of three independent mouse-derived preparations, and error bars indicate the standard deviation (n = 3 mice per workflow). For each mouse, the value was calculated as the mean of three technical measurements. Comparisons were descriptive, and no inferential statistical tests were performed.

Single-cell suspensions were evaluated using an automated fluorescence cell counter with AO/PI staining before library preparation. Both isolation workflows yielded sufficient numbers of viable cells for scRNA-seq (Figure 5A). Cell viability exceeded 90% in both preparations (Figure 5B), showing that viable single-cell suspensions suitable for downstream processing could be obtained without Ficoll-based purification. Subsequently, the suspensions were methanol-fixed and stored until scRNA-seq library preparation. All libraries analyzed in this study were generated from these fixed-cell suspensions.

### 3.2. QC of scRNA-seq Libraries

Approximately 24,000 viable cells were loaded for each library. The gradient-free library yielded 5568 initially identified cells, corresponding to an estimated capture efficiency of 23.2%, whereas the Ficoll-based library yielded 4358 cells, corresponding to 18.2%.

**Figure 6:**
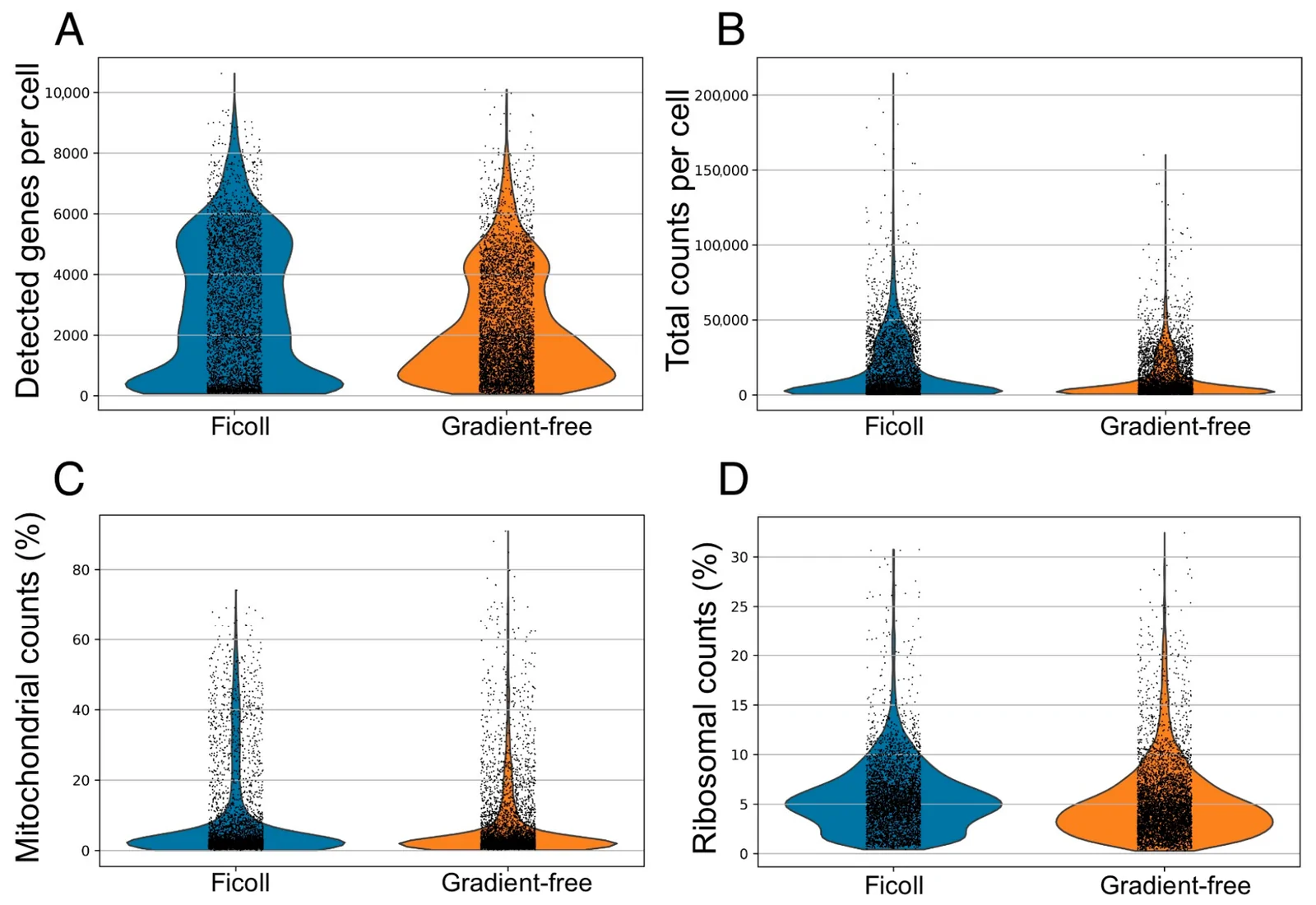
QC assessment of scRNA-seq libraries generated using the Ficoll-based and gradient-free islet isolation protocols. (**A**) Violin plot showing the number of detected genes per cell in the Ficoll-based and gradient-free scRNA-seq libraries. (**B**) Distribution of total transcript counts per cell between the two isolation protocols. (**C**) Percentage of mitochondrial transcript counts used as an indicator of cell quality and potential cell stress or damage. (**D**) Percentage of ribosomal transcript counts in cells obtained using the Ficoll-based and gradient-free protocols. Each point represents an individual cell. The plots provide a descriptive comparison of the principal QC metrics between the two libraries.

Before evaluating cell composition, the main QC parameters of the scRNA-seq libraries were examined. After QC filtering, 2659 cells from the gradient-free library and 2715 cells from the Ficoll-based library were retained for downstream analysis. The distributions of the number of detected genes per cell largely overlapped between the Ficoll-based and gradient-free libraries (Figure 6A). A similar pattern was observed for the total number of transcript counts per cell (Figure 6B). The proportion of mitochondrial transcripts remained low in both libraries (Figure 6C), while the proportion of ribosomal transcripts was also within a comparable range (Figure 6D). The distributions of these QC metrics broadly overlapped between the two libraries.

**Figure 7:**
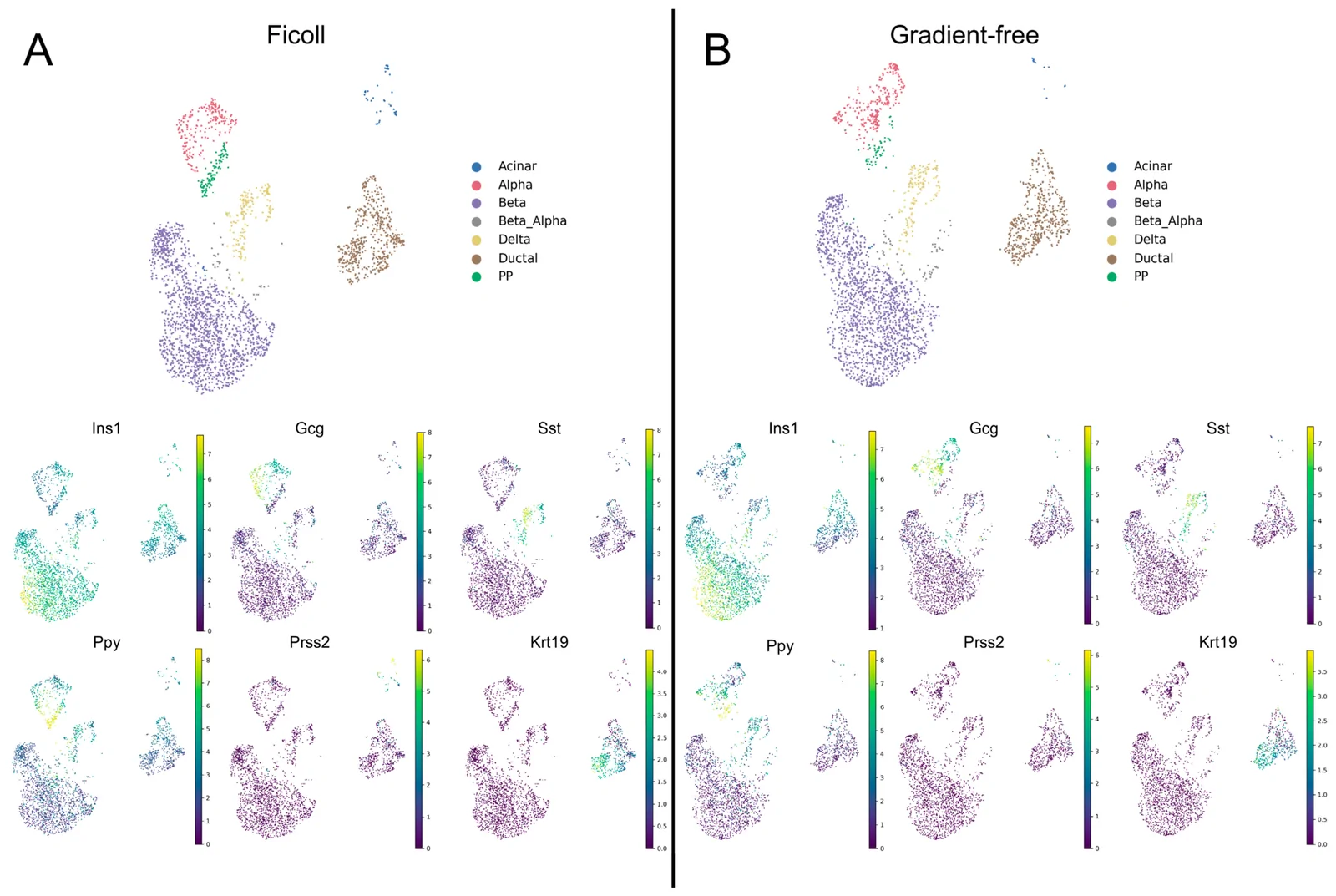
Cell identity validation of pancreatic islet scRNA-seq libraries generated using the Ficoll-based and gradient-free isolation workflows. (**A**) Ficoll-based library and (**B**) gradient-free library. In each panel, the upper UMAP shows annotated cell populations, including β-cells, α-cells, δ-cells, PP cells, ductal cells, acinar cells, and β/α-like cells. The lower feature plots show the expression of canonical marker genes in the corresponding library: Ins1 for β-cells, Gcg for α-cells, Sst for δ-cells, Ppy for PP cells, Prss2 for acinar cells, and Krt19 for ductal cells. Color intensity indicates normalized gene-expression levels.

After QC filtering and dimensionality reduction, separate UMAP projections were generated for each library. The major endocrine islet populations were identified in both datasets, including β-, α-, δ-, and PP cells, together with a small population of β/α-like cells. Acinar and ductal populations were also detected (Figure 7). Cell-type annotation was supported by the expression of canonical genes: *Ins1* for β-cells, *Gcg* for α-cells, *Sst* for δ-cells, *Ppy* for PP cells, *Prss2* for acinar cells, and *Krt19* for ductal cells (Figure 7).

**Figure 8:**
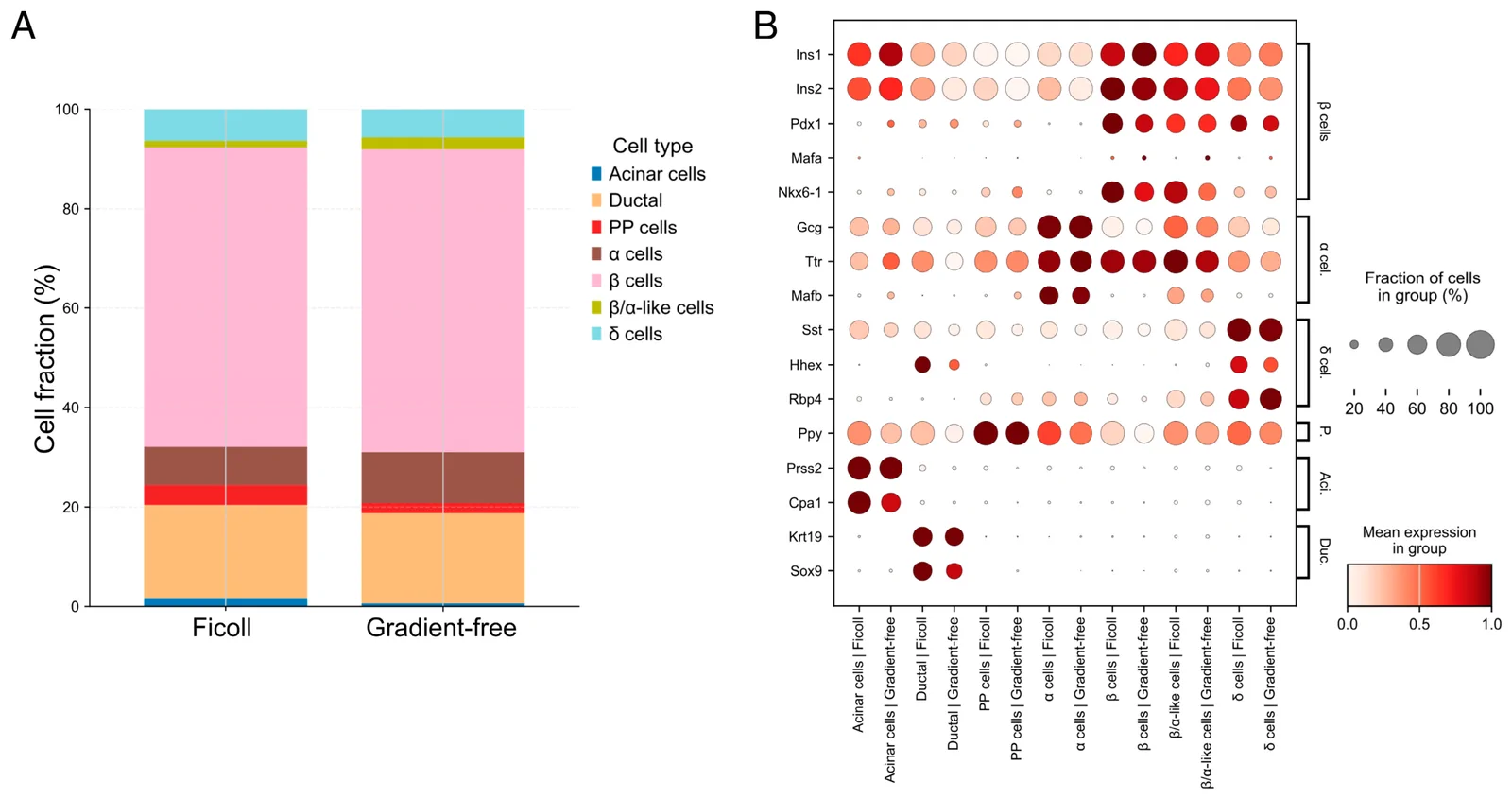
Comparison of cell composition and marker gene expression between the Ficoll-based and gradient-free islet isolation workflows. (**A**) Relative cell composition of the scRNA-seq libraries. Bars show the fraction of each annotated population among all analyzed cells. (**B**) Dot plot of canonical marker gene expression across annotated cell populations and isolation protocols. Dot size represents the fraction of cells expressing each gene, while color intensity indicates the scaled mean expression within the corresponding sample.

To quantify library composition, the proportion of each annotated population was calculated. β-cells were the predominant cell type in both libraries and accounted for approximately 60% of all analyzed cells. Ductal cells represented approximately 18% of each library. α-cells were slightly more abundant in the gradient-free library, whereas PP and acinar cells were more highly represented in the Ficoll-based library (Table 3; Figure 8A). An extended marker-gene panel further supported the annotation of the major populations: *Ins1*, *Ins2*, *Pdx1*, *Mafa*, and *Nkx6-1* in β-cells; *Gcg*, *Ttr*, and *Mafb* in α-cells; *Sst*, *Hhex*, and *Rbp4* in δ-cells; *Ppy* in PP cells; *Prss2* and *Cpa1* in acinar cells; and *Krt19* and *Sox9* in ductal cells (Figure 8B). The gradient-free library retained all major endocrine populations and showed a β-cell fraction similar to that observed in the Ficoll-based library.

### 3.3. Assessment of Stress-Associated Transcriptional Changes

To assess possible differences in cellular transcriptional state between the two scRNA-seq libraries, the expression of genes associated with cellular stress was analyzed. Because one pooled scRNA-seq library was generated for each workflow, all comparisons in this section are descriptive. Markers of the early stress response included *Fos*, *Fosb*, *Jun*, *Junb*, *Jund*, *Atf3*, *Egr1*, and *Dusp1*. Genes associated with the heat shock response (HSR) (*Hspa1a*, *Hspa1b*, *Hsp90aa1*, and *Hspb1*), ER stress (*Ddit3*, *Atf4*, and *Xbp1*), and oxidative stress (*Hmox1* and *Sod2*) were also evaluated.

**Figure 9:**
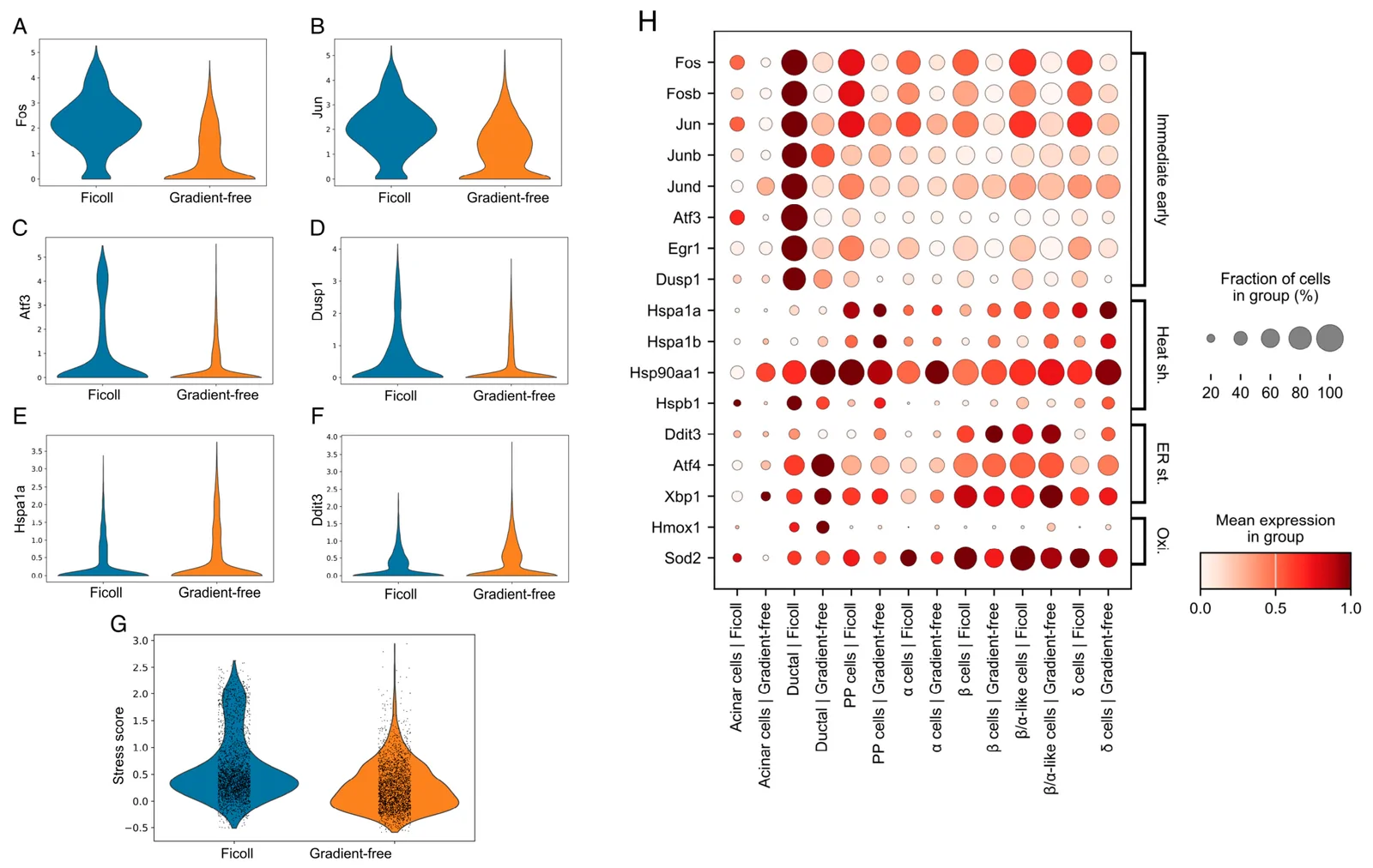
Stress-response gene expression in the Ficoll-based and gradient-free libraries. (**A**–**F**) Violin plots showing expression of selected stress-response genes: *Fos*, *Jun*, *Atf3*, *Dusp1*, *Hspa1a*, and *Ddit3*. (**G**) Distribution of the combined stress-response score calculated from the selected stress-associated gene panel. (**H**) Dot plot showing expression of stress-response genes across annotated cell populations and isolation workflows. Genes are grouped into IEG, HSR, ER stress, and oxidative stress categories. Dot size represents the fraction of cells expressing each gene, whereas color intensity indicates the scaled mean expression.

The most pronounced differences between the libraries were observed for immediate early response genes. Expression of *Fos*, *Jun*, *Atf3*, and *Dusp1* was generally higher in the pooled Ficoll-based library (Figure 9A–D). Dot-plot analysis showed that this pattern was particularly evident in ductal cells and in several endocrine populations (Figure 9H).

The expression patterns of heat shock- and ER stress-associated genes were less uniform. *Hspa1a* and *Ddit3* showed higher expression in the pooled gradient-free library (Figure 9E,F), whereas *Hsp90aa1*, *Xbp1*, and *Sod2* displayed broad, cell-type-specific expression without a consistent direction of change between the two libraries (Figure 9H).

A similar overall pattern was observed for the combined stress-response score calculated from the combined panel of stress-associated genes. The median score was 0.431 in the Ficoll-based library and 0.188 in the gradient-free library, while the corresponding mean values were 0.616 and 0.263, respectively (Figure 9G). Thus, the combined score was lower int the pooled gradient-free library despite higher expression of selected heat shock- and ER stress-associated genes. This comparison is descriptive and does not establish a method-specific effect.

Dot-plot analysis showed cell-type-specific variation in stress-associated gene expression. The most consistent differences between the two pooled libraries were observed for immediate early genes (IEGs), whereas HSR-, ER stress-, and oxidative stress-associated genes showed more variable expression across cell populations (Figure 9H).

## 4. Discussion

A key practical finding of this study is that density-gradient purification could be omitted while still obtaining viable single-cell suspensions suitable for scRNA-seq library preparation. The gradient-free workflow eliminated gradient assembly, interphase collection, large-volume dilution, and two subsequent washes. This shortened the estimated overall processing time by approximately 35 min, including 21.5 min of centrifugation. This finding is consistent with previous evidence that density-gradient separation is not essential for obtaining mouse islets of sufficient quality for downstream analysis. Ramírez-Domínguez and Castaño achieved effective purification using filtration rather than a density-gradient protocol [21]. In the present protocol (Figure 1), workflow simplification was achieved through controlled digestion and stereomicroscope-guided manual selection. A side-by-side comparison of processing times for the two workflows is provided in Table 4. Visual assessment allowed digestion to be stopped once adequate tissue fragmentation had been reached, while manual picking limited the need for additional centrifugation and transfer steps. These features may be particularly relevant when processing islets from diabetic animals, which may already be susceptible to metabolic and procedural stress [22].

Sequencing data provided an additional assessment of whether simplification of the isolation procedure was compatible with downstream transcriptomic analysis. Both libraries met the applied QC criteria, supporting the technical suitability of material obtained using the gradient-free workflow for downstream scRNA-seq analysis (Figure 6). Despite differences in sequencing depth and saturation, other sequencing and mapping quality metrics were broadly similar between the libraries (Table 1). Unequal sequencing depth was nevertheless considered when interpreting gene-expression and stress-response differences.

These metrics, however, describe library quality and cannot exclude more subtle processing-associated changes in cellular transcriptional state. Such changes may arise at several stages of sample preparation. Van den Brink et al. showed that tissue dissociation itself can generate transcriptional states that were absent from the original tissue in vivo [23]. Enzymatic digestion is therefore likely to be as important as the subsequent purification step. In the present protocol, digestion was monitored visually and terminated after adequate tissue fragmentation, thereby limiting unnecessary collagenase exposure. Density-gradient purification may introduce additional variability, as differences in gradient composition have previously been associated with changes in islet recovery and function [24,25].

Major endocrine populations were represented in both libraries, and β-cells remained the predominant cell type. The broadly similar distribution of endocrine populations suggests that omission of density-gradient purification was not accompanied by an evident selective loss of the endocrine compartment. However, this comparison should be interpreted descriptively because this study did not include matched biological replicates for the two workflows. Cell proportions in scRNA-seq libraries should not be considered direct estimates of the original tissue composition because they are affected by dissociation efficiency, differential cell survival, capture probability, and QC filtering.

Both protocols retained residual ductal and acinar populations, indicating that neither produced an exclusively endocrine preparation. The similar representation of ductal cells in the two libraries nevertheless suggests that this residual component was not specific to manual gradient-free purification. For the present application, retention of the major endocrine populations and recovery of sufficient viable cells were considered more important than complete removal of all non-endocrine cells. However, the extent of residual exocrine contamination may partly depend on the efficiency and consistency of manual islet selection. Manual islet selection is inherently operator-dependent, as differences in experience and technique can influence the consistency of islet identification and purification. This source of pre-analytical variability should be taken into account when establishing isolation workflows, particularly for sensitive downstream applications such as scRNA-seq. Selection may also become biased if operators preferentially collect large, intact, or visually prominent islets while overlooking smaller or partially disrupted structures. To reduce this effect, manual selection should follow predefined criteria and include all visually identifiable islets rather than only those with an “ideal” morphology. Standardized training and clearly defined selection criteria are therefore important for improving consistency across operators.

Transcriptional differences between the libraries were most evident in the immediate early response. The gradient-free library showed a lower combined stress-response score and lower expression of several immediate early genes, whereas heat shock-, ER-, and oxidative stress-associated genes showed no consistent direction of change. These findings therefore indicate differences in selected processing-associated transcriptional responses rather than a general reduction in cellular stress.

Importantly, both workflows included stereomicroscope-guided manual selection with repeated aspiration, transfer, and ex vivo handling, which may themselves induce mechanical or transcriptional stress. Thus, omission of Ficoll reduces Ficoll-specific processing steps but does not demonstrate a reduction in total cellular stress. The lower immediate early response in the pooled gradient-free library should therefore be regarded as a descriptive difference between the two preparations.

Immediate early gene expression may reflect both the biological state of the cells and their response to enzymatic and mechanical processing. O’Flanagan et al. similarly identified a reproducible transcriptional stress program induced by standard enzymatic dissociation [26]. These observations reinforce the need to distinguish processing-associated responses from stable biological differences when interpreting single-cell datasets.

The principal limitation is the absence of matched biological replicates for the two workflows. Each analyzed library was generated from pooled material, and variation among individual animals could therefore not be assessed. Inter-operator reproducibility was not assessed because all isolations were performed by a single operator. Consequently, differences in cell composition and transcriptional state should be regarded as descriptive rather than as evidence of a method-specific effect. In addition, islet yield per pancreas and pre-dissociation purity were not prospectively quantified. Although these parameters are useful for benchmarking isolation performance, islet number alone does not directly reflect the amount of material available for scRNA-seq because individual mouse islets vary in size and cell content; viable cell recovery after dissociation was therefore used as the primary input-related measure. Functional properties of the isolated islets were also not assessed, and the present data therefore do not establish preservation of glucose-responsive endocrine function. A direct comparison using several independently prepared, matched libraries would be required to determine whether the gradient-free workflow reproducibly affects cell recovery, cellular composition, or processing-associated transcriptional responses.

## 5. Conclusions

This study describes a gradient-free workflow for pancreatic islet isolation and single-cell dissociation from diabetic BKS.Cg-Dock7^m^ +/+ Lepr^db^/J mice. Omitting Ficoll-based purification eliminated gradient preparation, interphase recovery, and three centrifugation steps, shortening the estimated overall processing time by approximately 35 min, including 21.5 min of centrifugation. The workflow yielded viable single-cell suspensions suitable for scRNA-seq, and the pooled gradient-free library met the applied QC criteria and contained the major endocrine cell populations. These findings support the gradient-free workflow as a feasible alternative to Ficoll-based purification, without establishing superiority of one isolation method over the other. Because only one pooled scRNA-seq library was generated for each workflow, the observed differences in cell composition and stress-associated gene expression remain descriptive and cannot be attributed to the isolation method. Independent, matched library preparations are needed to establish reproducibility and determine whether the gradient-free workflow reproducibly affects cell recovery, cellular composition or processing-associated transcriptional responses.

## Figures and Tables

**Table 1:** scRNA-seq sequencing metrics.

QC Stage	Gradient-Free Workflow	Ficoll-Based Workflow
Mean reads per cell	46,913	87,251
Sequencing saturation	32.82%	46.59%
Fraction reads in cells	81.23%	76.20%
Valid barcodes	94.46%	95.71%
Q30 bases in barcode	94.63%	94.75%
Q30 bases in UMI	90.50%	90.59%
Reads mapped to a genome	94.77%	95.34%
Reads mapped confidently to a genome	88.21%	89.47%
Total genes detected	21,446	20,459

**Table 2:** Summary of scRNA-seq quality-control and cell-retention metrics.

QC Stage	Gradient-Free Workflow	Ficoll-Based Workflow
Initially identified cells	5568	4358
Retained after gene/count/mt QC	4430	3278
Scrublet-predicted doublets removed	188	109
All cells retained after doublet removal	4242	3169
Final endocrine/exocrine cells used for downstream analysis	2659	2715

**Table 3:** Relative cell composition (%) of scRNA-seq libraries generated using the Ficoll-based and gradient-free protocols.

	Β-Cells	α-Cells	δ-Cells	PP Cells	Ductal	β/α-like Cells	Acinar Cells
**Ficoll**	60.3	7.7	6.4	4.0	18.8	1.3	1.7
**Gradient-free**	60.9	10.3	5.7	2.0	18.2	2.4	0.6

**Table 4:** Comparison of processing times for the gradient-free and Ficoll-based islet isolation workflows.

Processing Step	Time, Gradient-Free Workflow	Time, Ficoll-Based Workflow
2.2 Pancreatic perfusion	10–15 min per mouse	10–15 min per mouse
2.3 Primary pancreatic digestion	up to 15 min; with monitoring every 5 min	up to 15 min; with monitoring every 5 min
2.4 Termination of digestion + primary washing	5 min, centrifugation for 2 min	5 min, centrifugation for 2 min
2.5 Removal of large fragments + additional washing	5 min, centrifugation for 2 min	5 min, centrifugation for 2 min
2.6 Ficoll purification	Omitted	35 min, including 21.5 min of centrifugation and ~15 min of Ficoll-specific handling.
2.7 Manual islet selection	15–30 min per dish	15–30 min per dish
2.8 Preparation for dissociation	5 min, centrifugation for 3 min	5 min, centrifugation for 3 min
2.9 Accutase dissociation	10–15 min, pipetting every 3 min	10–15 min, pipetting every 3 min
2.10 Washing/concentration after dissociation	5 min, centrifugation for 3 min	5 min, centrifugation for 3 min
2.11 QC before scRNA-seq	5–15 min per sample	5–15 min per sample
2.12 Optional methanol fixation	40 min, including ≥30 min at −20 °C	40 min, including ≥30 min at −20 °C
2.13 Washing fixed cells	10 min	10 min
2.14 Resuspension after fixation	5–15 min	5–15 min
Estimated processing time before methanol fixation	75–110 min	110–145 min

## Data Availability

Raw and processed sequencing data generated in this study have been deposited at GEO NCBI (accession number: GSE347182) and are publicly available as of the date of publication. All original codes have been deposited at Github (https://github.com/RuchkoEvgeny/Gradient-Free-Isolation-of-Murine-Pancreatic-Islets-for-Single-Cell-RNA-Sequencing, (accessed on 15 September 2026)) and are publicly available as of the date of publication.

## Abbreviations

The following abbreviations are used in this manuscript:

scRNA-seq	Single-cell RNA sequencing
QC	Quality control
ER	Endoplasmic reticulum
BSA	Bovine serum albumin
RPMI 1640	Roswell Park Memorial Institute 1640 medium
HBSS	Hanks’ balanced salt solution
PBS	Phosphate-buffered saline
AO/PI	Acridine orange/propidium iodide
UMAP	Uniform Manifold Approximation and Projection
SD	Standard deviation
HSR	Heat shock response
IEGs	Immediate early genes

## Appendix A

**Table A1:** Reagents and materials used for pancreatic islet isolation, single-cell preparation, and scRNA-seq library construction.

Reagent or Material	Manufacturer	Working Concentration or Application
Collagenase type I	Worthington Bio-chemical Corpora-tion, Lakewood, NJ, USA	3 mg/mL in DPBS; used for intraductal pancreatic perfusion and tissue digestion
Collagenase type II	Worthington Biochemical Corporation, Lakewood, NJ, USA	3 mg/mL in DPBS; used for intraductal pancreatic perfusion and tissue digestion
Dulbecco’s phosphate-buffered saline	PanEco, Moscow, Russia	Solvent for the collagenase solution and a cold medium for pancreatic tissue collection
DNase I	Thermo Fisher Scientific, Waltham, MA, USA	100 U/mL in a collagenase solution and post-digestion wash buffers
Hanks’ balanced salt solution	PanEco, Moscow, Russia	Supplemented with 0.3% BSA and 100 U/mL DNase I for washing and manual islet selection
Bovine serum albumin	Sigma-Aldrich, St. Louis, MO, USA	0.3% in HBSS and RPMI 1640; 0.1% or 0.04% in PBS during single-cell preparation
Tribromoethanol (Avertin)	Labtech Ltd., Moscow, Russia	Administered intraperitoneally at a dose of 250 mg/kg. A 2.5% working solution was used, corresponding to an injection volume of 10 µL/g body weight.
RPMI 1640 medium	PanEco, Moscow, Russia	Used for Ficoll gradient layering, dilution, washing, and resuspension
Ficoll, density 1.100 g/mL	PanEco, Moscow, Russia	Lower-density-gradient layer used in the Ficoll-based workflow
Ficoll, density 1.077 g/mL	PanEco, Moscow, Russia	Upper-density-gradient layer used in the Ficoll-based workflow
Phosphate-buffered saline without Ca^2+^ and Mg^2+^	PanEco, Moscow, Russia	Used for preparation, washing, filtration, fixation, and resuspension of single-cell suspensions
Accutase containing EDTA	STEMCELL Technologies, Vancouver, BC, Canada	1 mL per ≤300 islets; used for enzymatic dissociation at 37 °C
Methanol, molecular biology grade, ≥99.8%	Chemmed, Moscow, Russia	Four volumes added to one volume of cell suspension for fixation
Saline–sodium citrate buffer	Sigma-Aldrich, St. Louis, MO, USA	3× final concentration in fixed-cell wash buffer
Triton X-100	AppliChem GmbH, Darmstadt, Germany	0.1% in fixed-cell wash buffer
RiboCare RNase inhibitor	Evrogen, Moscow, Russia	1 U/µL final concentration in fixed-cell wash and resuspension buffers
Acridine Orange	Sigma-Aldrich, St. Louis, MO, USA	Used with Propidium Iodide for fluorescent cell counting
Propidium Iodide	Wuhan Servicebio Technology Co., Ltd., Wuhan, China	Used with Acridine Orange for viability assessment
SeekOne^®^ Single-Cell 3′ Gene Expression Library Preparation Kit, 8 reactions	Beijing SeekGene BioSciences Co., Ltd., Beijing, China	Used for preparation of whole-transcriptome scRNA-seq libraries
